# Overcoming the Recalcitrance for the Conversion of Kenaf Pulp to Glucose via Microwave-Assisted Pre-Treatment Processes

**DOI:** 10.3390/ijms12031451

**Published:** 2011-02-24

**Authors:** Beng Guat Ooi, Ashley L. Rambo, Miguel A. Hurtado

**Affiliations:** 1 Department of Chemistry, Middle Tennessee State University, P.O. Box 68, Murfreesboro, TN 37132, USA; E-Mail: mah5a@mtmail.mtsu.edu; 2 East Tennessee State University, Johnson City, TN 37614, USA; E-Mail: bcca13@yahoo.com

**Keywords:** cellulose, crystalline, lignin, cellulase, ethanol, pre-treatment, glucose, kenaf fiber, Avicel, microwave

## Abstract

This study evaluates the pre-treatment of cellulose from kenaf plant to yield sugar precursors for the production of ethanol or butanol for use as biofuel additives. In order to convert the crystalline cellulosic form to the amorphous form that can undergo enzymatic hydrolysis of the glycosidic bond to yield sugars, kenaf pulp samples were subjected to two different pre-treatment processes. In the acid pre-treatment, the pulp samples were treated with 37.5% hydrochloric acid in the presence of FeCl_3_ at 50 °C or 90 °C whereas in the alkaline method, the pulp samples were treated with 25% sodium hydroxide at room temperature and with 2% or 5% sodium hydroxide at 50 °C. Microwave-assisted NaOH-treatment of the cellulose was also investigated and demonstrated to be capable of producing high glucose yield without adverse environmental impact by circumventing the use of large amounts of concentrated acids *i.e.*, 83–85% phosphoric acid employed in most digestion processes. The treated samples were digested with the cellulase enzyme from *Trichoderma reesei*. The amount of glucose produced was quantified using the Quantichrom^™^ glucose bioassay for assessing the efficiency of glucose production for each of the treatment processes. The microwave-assisted alkaline pre-treatment processes conducted at 50 °C were found to be the most effective in the conversion of the crystalline cellulose to the amorphous form based on the significantly higher yields of sugar produced by enzymatic hydrolysis compared to the untreated sample.

## 1. Introduction

Kenaf (*Hibiscus cannabinus*) is a woody plant cited by the researchers at the United States Department of Agriculture (USDA) as one of the most promising crops for industrial development [1]. The crop is an excellent choice for soil and water remediation as well as a top producer of bioenergy feedstock. Currently, it is grown mostly for the purpose of yielding cellulosic fibers for pulp and paper industry. Researchers are working on converting the by-products from the paper processing into fertilizer and feed binder. The kenaf fiber is superior to wood fiber for paper, textiles, and pressed wood materials. The foliage provides a high quality forage resource while the remainder of the plant can be used to produce animal bedding and litter with high absorption capacity and odor control media. The crop is disease-resistant and does not require herbicide, pesticide, or heavy fertilizer inputs [2,3]. Therefore, the biomass production is cost effective and ecologically sustainable. The kenaf cellulosic materials could be a renewable source of fermentable sugars for the production of ethanol and *n*-butanol fuel additives. To make this economically viable, the cellulosic materials must be converted to a form that can be degraded by chemical or biological processes.

The cellulose of plant cell wall contains an abundance of glucose monomers bonded via β(1–4)-glycosidic linkages. The polysaccharide is in the form of a straight chain, which allows its hydroxyl groups to undergo both inter-molecular and intra-molecular hydrogen bonding. Therefore, cellulose has a highly crystalline structure making it difficult for the cellulase enzyme to access and hydrolyze the linkages to release the glucose units [4,5]. The resistance of the cellulose structure to cellulase digestion is due to the degree of crystallinity as well as the shielding by the hemicellulose and lignin barriers. Cellulase, which function through the synergistic action of three of its components, *i.e.*, the exo-β-glucanase, endo-β-glucanase, and β-glucosidase readily access and hydrolyze the β(1–4)-glycosidic linkages in amorphous or highly decrystallized cellulose. Various pre-treatment [5–7] techniques including mercerization [8,9] and dissolution [10,11] procedures have been used to transform the natural cellulose (CI) structure of wood, cotton linter, and sugar cane bagasse to the cellulose II (CII) and amorphous form. In these studies, the degrees of transformation were evaluated using the wide-angle X-ray scattering (WAXS) technique [12,13], the ^13^C NMR spectroscopy [14,15], or the ^13^C-CP/MAS NMR spectroscopy techniques [16], all of which are effective for monitoring changes in cellulose crystals. Other techniques useful for studying lignocellulose materials include the conventional dispersive Raman spectroscopy [17,18] and the near-infrared Fourier Transform Raman spectroscopy [19,20].

A recent method developed by Cellulose Science International (CSI) for decrystallization of corn stover reportedly result in 69% glucose conversion, which is almost double the usual 36% conversion after 10 hours of enzymatic hydrolysis [21]. The procedure requires two stages including initial enzyme hydrolysis, which result in about 40% conversion followed by the decrystallization process for the residual cellulose that is recalcitrant toward the initial hydrolysis. Another two-step mild alkaline/oxidative pre-treatment procedure at low temperature by conventional heating took a total of 48 hours to successfully lower the hemicellulose content of wheat straw [22]. Zhu and co-workers accelerated the heating process to 60 minutes using domestic kitchen microwave coupled with alkali [23] or a combination of acid/alkali/H_2_O_2_ [24] pre-treatment and showed that the microwave/acid/alkali/H_2_O_2_ procedure was most effective at removing most of the hemicellulose barrier from rice straw resulting in the highest glucose yield. The present study investigates the effectiveness of cellulose decrystallization in kenaf pulp using NaOH and ethanol pre-treatment with a microwave reactor versus an acid-catalyzed method. The outcome was evaluated by the amount of glucose produced after enzymatic hydrolysis by the cellulase enzyme from *Trichoderma reesei*.

## 2. Experimental Section

### 2.1. NaOH Pre-Treatment of Avicel and Kenaf Using Microwave

Microcrystalline cellulose or Avicel PH-101 from Fluka Biochemika was purchased from Sigma-Aldrich, St. Louis, MO, USA. The Avicel powder was treated using a modified mercerization method [8] with and without exposure to microwave energy. A quantity of 0.6 g Avicel powder was treated with 25% (w/w) sodium hydroxide in 10% (w/w) ethanol (Pharmco-AAPER, Shelbyville, TN, USA) co-solvent at room temperature for 45 minutes. In one pre-treatment procedure, the ethanol co-solvent and the NaOH base were added to the kenaf pulp for a total treatment time of 45 minutes. In another, the ethanol was added after 30 minutes of initial NaOH pre-treatment and mixed for an additional 15 minutes at room temperature. The Discover^™^ microwave reactor (CEM Corporation, Matthews, NC, USA) was programmed with a temperature setting of 22 °C at 300 psi for a holding time of 30 minutes, with the stirring rate set on “high”; the maximum microwave power was set at 5 watts and the actual power recorded was at 1 watt. To facilitate the decrystallization, the NaOH/ethanol-treated samples were washed repeatedly with water containing 10% (w/w) ethanol until pH 7. The “Walseth” was made by treating the Avicel powder with cold 85% phosphoric acid according to the method described by T. W. Jeffries [25]. The “Walseth” and untreated Avicel were used as controls.

Kenaf or *Hibiscus cannabinus* used in this study was grown locally. The cores of the stalks were ground into a fine pulp after the bark was removed. The amount corresponding to 0.6 g of kenaf pulp was soaked in approximately 25–28% (w/w) sodium hydroxide for 30 minutes at room temperature before adding the 10% (w/w) ethanol co-solvent. The experiments were carried out either with or without applying microwave energy in order to assess the contribution of microwave to improving yields. The pulp in the NaOH-ethanol solvent was mixed continuously for 15 minutes. Pre-treatment of kenaf pulp was repeated with 2% (w/w) and 5% (w/w) sodium hydroxide at 50 °C for 30 minutes without and with microwave followed by the 10% ethanol co-solvent treatment. The microwave power setting for the 50 °C pre-treatment was 50 watts but the recorded power ranged from 7–9 watts. The microwave pressure setting was at 300 psi, hold time at 30 minutes, and stirring set on “high”. The microwave temperature was monitored by an infrared sensor so that the microwave power could be automatically adjusted to maintain a constant temperature. Additional regulation was achieved by directing a stream of cold air into the chamber that housed the sample tube. Pre-treated kenaf samples were washed repeatedly with water containing ethanol until pH 7 and stored at 4 °C. Untreated kenaf pulp and pulp treated with cold 85% phosphoric acid [25] were used as controls.

### 2.2. Acid Pre-Treatment of Kenaf

Kenaf pulp samples were subjected to different pre-treatment conditions (T1–T3) as described in the Table 1.

Treatments were carried out at 50 °C for 3 hours and 90 °C for 15 minutes. Glycerol was used in place of deionized water (dH_2_O) for samples treated at 90 °C to prevent acid-induced charring at higher temperatures. Prior to cellulase treatment, samples were washed several times with deionized water until pH 7 was obtained and stored at 4 °C.

### 2.3. Enzyme Hydrolysis and Glucose Assay

Pre-treated pulp and Avicel equivalent to 25 mg dry weight were re-suspended in 0.05 M of sodium acetate buffer at pH 5.2 to which 50 units of cellulase enzyme from *Trichoderma reesei* was added to a final volume of 5 mL after equilibrating the samples for 10 minutes in a water bath at 50 °C. Samples for glucose analysis were collected at 3.0, 6.0, and 24 hours after adding the cellulase. The undigested Avicel and kenaf fibers were pelleted and the supernatant solution was assayed for glucose. The amount of glucose produced was quantified using the Quantichrom^™^ glucose bioassay (Thermo Fisher Scientific Inc., Waltham, MA) for assessing the efficiency of glucose production for each treatment method. The method involved boiling the samples in a reagent for 8 minutes before being cooled for 4 minutes prior to measuring the optical density at 630 nm with the Hitachi U-2000 UV-Vis spectrophotometer. The digestion was carried out for triplicate samples with each sample being subjected to bioassays for glucose levels three times.

### 2.4. Raman Microscopy Analyses

Moist pre-treated kenaf fiber samples were sandwiched between two Grade-4 mica sheets of 15 × 15 × 0.15 mm (SPI supplies, West Chester, PA, USA). The degree of delignification and decrystallization of the kenaf fibers was evaluated using the ProRaman-L Raman spectrometer (Enwave Optronics, Irwine, CA, USA) that was coupled to an Olympus CX31 microscope. The spectrometer has a 785-nm laser for sample excitation and a maximum laser power of 300–400 mW. The kenaf fiber was imaged and probed using a 40x microscope objective (MEIJI Techno, Japan) with a finite tube length objective (F) of 200 mm and a working distance of 0.5 mm. The Raman spectral measurements were acquired with the integration time of 130–150 seconds.

## 3. Results and Discussion

### 3.1. Alkaline Pre-treatment of Avicel

The alkaline pre-treatment that was normally used in mercerization processes [8] with 10% ethanol co-solvent [9,21] resulted in relatively high glucose yields for both the Avicel (Table 2) and kenaf pulp (Table 3a and 3b) when compared with the untreated samples. The cellulase readily digested the NaOH-treated Avicel samples with about 85–89% conversion to glucose after 3 hours of pre-treatment. The “Walseth” or phosphoric acid treated Avicel control had about 94% conversion to glucose completed by 3 hours of digestion. Compared to untreated Avicel, the relative glucose yield for the “Walseth” and the 25% NaOH-treated samples were about 2.7 times and 2.4 times the yield of the untreated sample, respectively. The digestion was almost complete with about 96–100% glucose conversion after 6 hours of enzymatic digestion for all the samples except for the untreated Avicel with only 71% conversion. The best three experimental measurements of glucose production for the NaOH-treated samples produced relative yields that ranged from 2.0–2.1 times the yield of the untreated sample. These values were comparable with the 2.1 times relative glucose yield of the “Walseth” sample after 6 hours of enzyme digestion. Prolonging the digestion time until 24 hours gave more time for the enzyme to penetrate and access the structure of the untreated samples. The 25% sodium hydroxide pre-treatment carried out at room temperature was conducive to disrupting the crystalline structure of Avicel that the additional treatment with low microwave energy was not necessary and did not result in any significant improvement in sugar yields. In fact, the difficulty in maintaining a low temperature during the microwave treatment may have resulted in the loss of the ethanol co-solvent and hence, lower sugar yields for the Avicel sample with the ethanol present during the microwave process (Table 2). This is consistent with previous findings that the ethanol co-solvent is important for maintaining the stability of the sample state of decrystallinity [21].

### 3.2. Alkaline Pre-Treatment of Kenaf Pulp

**Figure 1.**
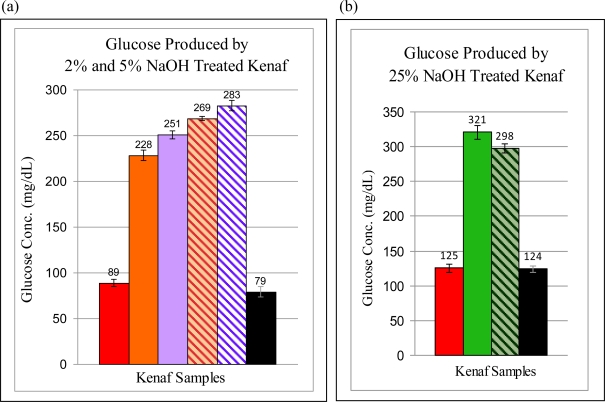
The Effects of Different NaOH Treatment Conditions on the Digestibility of Kenaf Pulp. The base treatment was performed with different percentages of NaOH and temperatures under the conditions of without microwave (solid column) and with microwave power (striped column). The phosphoric acid treated kenaf ( 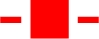) and the untreated kenaf ( 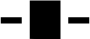) were the controls. The columns indicate the amount of glucose produced after 24 hours of enzymatic digestion of the pre-treated kenaf by cellulase. **(a)** Glucose produced by kenaf pre-treated with 2% ( 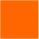) and 5% ( 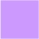) NaOH at 50°C without and with microwave energy. **(b)** Glucose produced by kenaf pre-treated with 25% ( 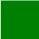) NaOH at room temperature without and with microwave energy.

Kenaf fibers were more difficult to digest compared to Avicel because of the hemicellulosic and lignin content [26]. Hence, the kenaf samples were digested for 24 hours with cellulase before quantifying the glucose yield (Table 3a and b). The kenaf pulp treated with 25% NaOH at room temperature without the use of microwave energy resulted in about 2.6 times the relative glucose yield of the untreated pulp after 24 hours of cellulase digestion (Table 3b) suggesting that the pre-treatment improved the accessibility of the pulp to the cellulase enzyme. Room temperature pre-treatment using low microwave energy gave similar results of glucose conversion, yielding about 2.4 times the amount of glucose obtained for the untreated pulp. However, the 2% and 5% NaOH pre-treatment conducted at a higher temperature of 50 °C using conventional heating significantly improved the relative glucose yield of the pulp to respectively, 2.9 and 3.2 times that of untreated pulp. This suggests that temperature enhances the pre-treatment processes even at low NaOH concentration. The increase in the microwave power to accommodate the increase in treatment temperature from 25 °C to 50 °C led to yields of glucose for the 2% and 5% NaOH-treated pulp that were 3.4 and 3.6 times, respectively, relative to the glucose concentration found in the untreated samples (Table 3a). In fact, the combination of microwave and 2% NaOH treatment resulted in a higher glucose yield than those obtained with conventional heating of the 2% NaOH and 5% NaOH-treated pulp (Figure 1a). This suggests that the presence of microwave energy can further aid in the decrystallization of the cellulose structure of the kenaf fibers. Using low power microwave energy did not result in the same benefit and therefore, the yield from the microwave-assisted 25% NaOH-treated pulp did not improve compared to pulp treated with 25% NaOH at 25 °C without microwave (Figure 1b). Unlike the “Walseth” Avicel, which produced the maximum glucose yield, the kenaf pulp treated with cold 85% phosphoric acid was no better than the untreated pulp in terms of glucose yield. The fibers were either more resistant to the acid pre-treatment at low temperature than Avicel because of the shielding by the hemicellulose and lignin or the decrystallization were not stable since the pre-treated fibers were washed in water instead of ethanol-water co-solvent. Since the digestibility of the cellulosic biomass in most biobased feedstocks are directly related to the hemicellulose removal [27], it is possible that the phosphoric acid pre-treatment method did not effectively remove most of the hemicellulose in the kenaf fibers and therefore the glucose yield was as low as that for untreated pulp.

### 3.3. Acid Pre-Treatment of Kenaf Pulp

The alkaline pre-treatment method produced a significantly higher relative glucose yield than the acid pre-treatment method. The HCl-FeCl_3_ catalyzed pre-treatment performed on kenaf pulp at a higher temperature of 90 °C for 15 minutes resulted in 1.4 times the glucose yield of the untreated sample whereas the same pre-treatment carried out at 50 °C for 3 hours resulted in a 1.2 times the glucose yield of the untreated sample (Table 4). These values are significantly lower than those obtained using the 2% and 5% NaOH-treatment method. A high relative yield of 1.8 times the glucose produced in untreated sample was obtained at the 90 °C with HCl only pre-treatment indicated that the FeCl_3_ catalyst was not required at very high temperature. To avoid any potentially undesirable side reactions of acid hydrolysis and the reduced decrystallinity effect on the cellulose at high temperature with longer reaction time as observed by Zhang *et al.* [10], the reaction time for the high temperature treatment was reduced to 15 minutes. In addition, glycerol, a less volatile solvent with a boiling point of 290 °C, was used in place of water for the high temperature pre-treatment process.

### 3.4. Raman Analyses of Kenaf Fibers

**Figure 2.**
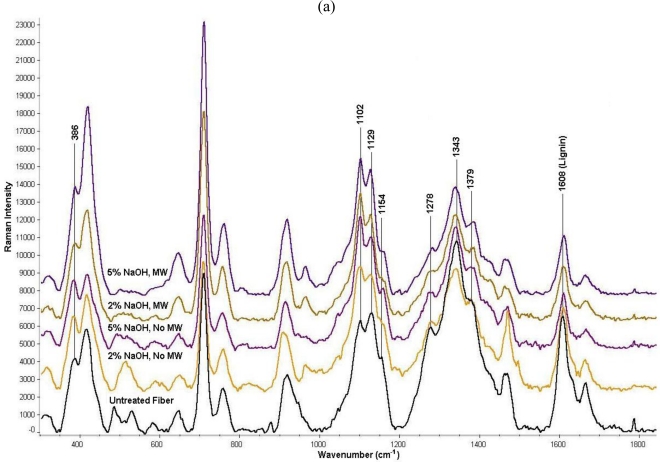

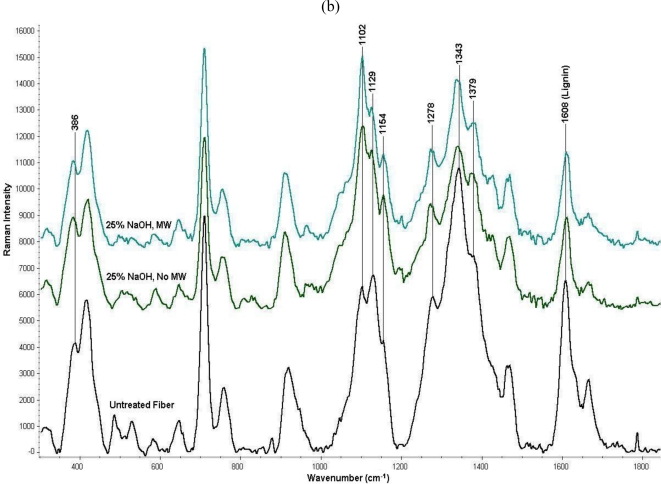
Raman Analysis of NaOH-Treated Kenaf Fiber. Individual kenaf fiber sandwiched between mica sheets was analyzed by a Raman microscope. The spectra for NaOH-treated fibers that were subjected to microwave radiation and no microwave radiation are labeled “MW” and “No MW”, respectively. The spectra are offset and shown on a common scale. **(a)** Raman spectra of 2% and 5% NaOH treated and untreated kenaf fibers that were subjected to reaction temperature of 50 °C. **(b)** Raman spectra of 25% NaOH treated and untreated kenaf fibers that were subjected to reaction at room temperature.

Raman spectral analysis of the NaOH-treated kenaf fibers showed that the lignin band due to the symmetric aryl ring stretching at 1608 cm^−1^ [20,28] was significantly reduced in the treated fibers relative to the untreated fibers (Figure 2a, b). It was also observed that the Raman peak at 2944 cm^−1^, which was attributed to the asymmetric CH stretch of OCH_3_ group associated with lignin, was significantly reduced in the spectra of all the NaOH-treated samples relative to the untreated fibers due to the loss of the lignin containing the methoxy groups [20]. Both observations of peak intensity trends at 1608 cm^−1^ and 2944 cm^−1^ suggest that the NaOH pre-treatment was able to remove some of the lignin shielding the cellulose from the hydrolytic action of the cellulase enzyme. Comparison of the Raman spectra in Figure 2a also shows that when the pre-treatment was conducted in the absence of microwave power, the use of 5% NaOH treatment was more effective in lowering the lignin content in the fiber compared to the experiment with 2% NaOH treatment. Furthermore, the application of microwave power seemed to elevate the rate of lignin removal in the 2% NaOH-treated fiber to the point that there was not any significant difference in the levels of lignin for the 2% NaOH-treated and 5% NaOH-treated fibers. The Raman peaks for the vibrational modes of the syringyl and guaiacyl moieties of lignin were observed for the kenaf pulp. However, the syringyl/guaiacyl ratio of lignin composition was not determined because their corresponding Raman peak separation is only 4–8 cm^−1^ for the most prominent ring stretching band and thus making it difficult for quantitative analysis due to the limited spectral resolution of the Raman microscope [29].

Peak shifts and peak distortions were observed for Raman spectral bands at 386 cm^−1^, 1102 cm^−1^, 1129 cm^−1^, 1154 cm^−1^, 1278 cm^−1^, 1343 cm^−1^, and 1379 cm^−1^ (Figure 2 a, b), all of which have been attributed to the cellulose vibrational bands mostly in the form of HCC and HCO bending and stretching modes according to several researchers [20,30]. The spectral differences imply that the NaOH treatment was not only significant in the delignification process but it was also crucial in causing the structural transformation of the cellulose that resulted in the higher glucose yields reported in Tables 3a and 3b. When the 25% NaOH treatment was performed for Avicel instead of kenaf fibers, the same type of spectral differences was also observed along with similar levels of improvement in glucose yields.

## 4. Conclusions

The NaOH pre-treatment method gave significantly higher glucose yield relative to untreated pulp compared to the HCl-FeCl_3_ catalyzed method (Tables 3 and 4). The major advantage of the NaOH pre-treatment is its ability to delignify and decrystallize the kenaf fiber at low NaOH concentrations of 2% or 5% sodium hydroxide at 50 °C, using either conventional heating or microwave radiation. The microwave-assisted pre-treatment of kenaf with 2% or 5% NaOH produced glucose yield in the range of 269–283 mg/dL, which corresponds to 3.4–3.6 times the glucose yield derived from untreated pulp whereas the same pre-treatment conducted with conventional heating produced glucose yield of 228–251 mg/dL that is equivalent to 2.9–3.2 times the glucose yield of untreated pulp. The microwave energy contributed to making the kenaf pulp more amenable to the cellulase activity of producing monosaccharides through the delignification and the decrystallization of the kenaf fibers. An ethanol co-solvent is required to stabilize the decrystallization processes. The acid method based on 37.5% HCl and the higher temperature of 90 °C, produced only about 1.8 times the glucose yield of untreated pulp. The NaOH method for cellulosic pre-treatment of kenaf pulp fulfills a major goal of biomass utilization in providing a treatment methodology that not only produces a significant increase in sugar yield but also has minimal environmental impact because this method precludes the need to use highly concentrated acids such as the 83% phosphoric acid used in other digestion processes [10]. For future work, it is important to further evaluate the efficiency of NaOH treatment for other types of cellulosic biomass such as agricultural wastes of corn stover, sugarcane bagasse, and rice husks.

The analysis of NaOH-treated kenaf fibers by Raman microscopy further corroborated the glucose bioassay data and supported the observation that the delignification process is critical to production of glucose from the lignocellulose in kenaf. The reduction in the intensity of the Raman band at 1608 cm^−1^ can potentially be utilized as a quantitative measure of the delignification efficiency for future research in cellulosic treatment. The promising results of NaOH pre-treatment for kenaf fibers should encourage further studies on the enzymatic hydrolysis of the pre-treated pulp and the fermentation of the sugar to be carried out simultaneously in the same reactor chamber using a specially selected *Saccharomyces cerevisiae* yeast strain [31]. Although the optical microscope used in this study does not allow a detailed study of the microstructural transformation of the kenaf fiber by the microwave irradiation, a prior publication has demonstrated using scanning electron microscopy that carboxymethylcellulose subjected to microwave radiation showed greater sample porosity [32]. This resulted in a larger surface area for the cellulase enzyme to yield a faster reaction rate for the samples with microwave pre-treatment relative to untreated samples. The microwave enhancement of catalytic efficiencies was also observed when the activities of xylanase and pectinase were evaluated with xylan from oat spelt and polygalacturonic acid from oranges [32].

## Figures and Tables

**Table 1.** Pre-treatment conditions for Kenaf samples.

**Samples**	**T1:HCl-FeCl_3_**	**T2: HCl only**	**T3:FeCl_3_ only**
Kenaf	1.5 g	1.5 g	1.5 g
dH_2_O	5.0 g	5.0 g	5.0 g
12 M HCl	3.0 mL	3.0 mL	3.0 mL dH_2_O
FeCl_3_	0.5 g	--------	0.5 g

**Table 2.** Glucose produced by NaOH-treated Avicel. Avicel was treated with 25% NaOH with and without microwave energy at room temperature. The 10% ethanol co-solvent may be present with the NaOH or added after the initial NaOH treatment. The amount of glucose produced after ^a^ 3.0, ^b^ 6.0, and ^c^ 24 hours of cellulase digestion of pre-treated Avicel was determined using the glucose bioassay. In order to correct for variations in cellulase activities during the experiment, the relative yield of glucose from each sample was compared with the glucose produced from the untreated Avicel control experiment conducted at the same time.

	**Control**	**No Microwave**		**Microwave**		**Control**
Samples	“Walseth” Avicel	NaOH − 30 min, EtOH − 15 min	NaOH + EtOH − 45 min	NaOH − 30 min, EtOH − 15 min	NaOH + EtOH − 45 min	Untreated Avicel
^a^.[Glucose] mg/dL	534 ± 16	480 ± 6	478 ± 12	481 ± 16	453 ± 8	200 ± 5
Relative Yield	2.7×	2.4×	2.4×	2.4×	2.3×	1.0×
^b^.[Glucose] mg/dL	552 ± 7	559 ± 13	542 ± 19	542 ± 8	499 ± 6	268 ± 3
Relative Yield	2.1×	2.1×	2.0×	2.0×	1.9×	1.0×
^c^.[Glucose] mg/dL	570 ± 34	559 ± 22	565 ± 19	551 ± 22	507 ± 8	376 ± 11
Relative Yield	1.5×	1.5×	1.5×	1.5×	1.4×	1.0×

**Table 3.** (**a**) and (**b**). Comparison of Glucose Levels Produced by NaOH-treated Kenaf under Different Experimental Conditions. Kenaf pulp was treated with different concentrations of NaOH either with or without the use of microwave energy for 30 minutes followed by 10% ethanol co-solvent pretreatment for an additional 15 minutes. The amount of glucose produced after 24 hours of digestion by cellulase of the pre-treated kenaf was determined. In order to correct for variations in enzyme activities when comparing glucose yields for various sets of experiments, relative yields of glucose production are calculated by dividing the glucose yields of treated samples by the glucose yields of the untreated kenaf sample.

(a)						
**Conditions**		**50 °C, 30 minutes**				
	**Control**	**Conventional Heating**		**Microwave Heating**		**Control**
Samples	PO_4_^3−^ Kenaf	2% NaOH	5% NaOH	2% NaOH	5% NaOH	Untreated Kenaf
[Glucose] mg/dL	89 ± 4	228 ± 6	251 ± 5	269 ± 2	283 ± 5	79 ± 6
Relative Yield	1.1×	2.9×	3.2×	3.4×	3.6×	1.0×

(b)				
**Conditions**		**Room Temperature, 30 minutes**		
	**Control**	**No Microwave**	**Microwave**	**Control**
Samples	PO_4_^3−^ Kenaf	25% NaOH	25% NaOH	Untreated Kenaf
[Glucose] mg/dL	125 ± 5	321 ± 10	298 ± 6	124 ± 4
Relative Yield	1.0×	2.6×	2.4×	1.0×

**Table 4.** Glucose Produced By Acid Pre-treated Kenaf. The amount of glucose produced after 24 hours of digestion by cellulase was determined. To avoid variations in enzyme activities, the yields of glucose for the treated samples are divided by the glucose yield for the control or untreated kenaf sample to give relative yields. The phosphoric acid treated kenaf was an additional control that was conducted at the same time with the batch of kenaf samples pre-treated for 0.25 hours at 90 °C.

**Treatment**	**50 °C for 3 Hours**				**Control**	**90 °C for 0.25 Hours**			
Samples	HCl–FeCl_3_	HCl	FeCl_3_	Untreated Kenaf	PO_4_^−3^ Kenaf	HCl–FeCl_3_	HCl	FeCl_3_	Untreated Kenaf
[Glucose] mg/dL	97 ± 2	89 ± 4	78 ± 3	81 ± 3	67 ± 4	94 ± 3	119 ± 5	58 ± 4	68 ± 4
Relative Yield	1.2×	1.1×	1.0×	1.0×	1.0×	1.4×	1.8×	0.9×	1.0×
